# Optimization of Controlled Water and Nitrogen Fertigation on Greenhouse Culture of* Capsicum annuum*

**DOI:** 10.1155/2018/9207181

**Published:** 2018-04-15

**Authors:** Youzhen Xiang, Haiyang Zou, Fucang Zhang, You Wu, Shicheng Yan, Xinyan Zhang, Jianke Tian, Shengcai Qiang, Haidong Wang, Hanmi Zhou

**Affiliations:** ^1^College of Water Resources and Architectural Engineering, Northwest A&F University, Yangling 712100, China; ^2^Key Laboratory of Agricultural Soil and Water Engineering in Arid and Semiarid Areas of Ministry of Education, Northwest A&F University, Yangling 712100, China; ^3^College of Agricultural Engineering, Henan University of Science and Technology, Luoyang 471003, China

## Abstract

This study investigated the effects of different combinations of irrigation and nitrogen levels on the growth of greenhouse sweet peppers, assessing yield, quality, water use efficiency (WUE), and partial factor productivity from applied N (PFPN). By using controlled drip irrigation, the optimal conditions for efficient, large-scale, high-yield, and high quality production of sweet peppers in Northwest China were determined. Using the local conventional irrigation and nitrogen regime as a control (105% ET_0_, N: 300 kg·hm^−2^), three alternative irrigation levels were also tested, at 90%, 75%, and 60% ET_0_. These were combined with nitrogen levels at 100%, as the control, and 75%, 50%, and 25%, resulting in 16 combination treatments. The results show that different supplies of water and nitrogen nutrition had a significant impact on the growth, yield, WUE, PFPN, and quality of fruit. The treatments of W_0.90_N_0.75_, W_0.90_N_0.50_, W_0.75_N_0.75_, and W_0.75_N_0.50_ can better maintain the “source-sink” relationship of peppers. They increased the economic yield, WUE, and PFPN. A principal component analysis was performed to evaluate indicators of fruit quality, revealing that the treatment of W_0.75_N_0.50_ resulted in the best fruit quality. For greenhouse sweet peppers produced in Northwest China, the combination of W_0.90_N_0.75_ resulted in the highest economic yield of 34.85 kg·hm^−2^. The combination of W_0.75_N_0.75_ had the highest WUE of 16.50 kg·m^−3^. The W_0.75_N_0.50_ combination treatment had the highest fruit quality score. For sustainable ecological development and in view of limited water resources in the area, we recommend the W_0.75_N_0.50_ combination treatment, since it could obtain the optimal fruit quality, while its economic yield and WUE were 9% and 4% less than the maximum, respectively. This study provides a theoretical basis for the optimal management of water and nitrogen during production of greenhouse sweet peppers in Northwest China.

## 1. Introduction

The ever-increasing imbalance between supply and demand of agricultural water has forced adjustments of modern agricultural irrigation practices [1]. Crop yields generally benefit from short growth cycles and the selection of high-yield varieties that undergo rapid development, strategies that have effectively increased the income of farmers. However, in order to maximize yield, excessive irrigation and fertilization are often required, resulting in overuse and serious runoff of fertilizer. Other negative effects are more frequent plant diseases and pests [2], a reduction of product quality, less efficient water and fertilizer utilization [3], and damage to local soil and water environments [4].

Water and nitrogen are critical factors determining crop yield and quality [5]. Appropriate irrigation technologies and nitrogen application strategies can lead to high yields and growth efficiencies of crops. Compared to conventional furrow irrigation, fertigation (the injection of fertilizer solution into the soil) effectively reduces surface runoff, evaporation between plants, and deep percolation [6]. At the same time, the drip irrigation technology with small flow can lower the downward migration rate of nitrogen into the soil and reduce nutrient loss [7]. As a result, a suitable nutrient microenvironment in the crop root zone can be achieved, promoting absorption of nutrients into the crops.

*C. annuum* (L. var. grossum), a member of the Solanaceae, produces fruits with a high nutritional value. Its effective culture is strongly dependent on the environment [8], as the plant requires a constant supply of water and nutrients [9]. The availability of nitrogen is an important determinant of crop yield and quality, by directly affecting photosynthesis and the accumulation, transfer, and distribution of biomass [10]. Previously, the studies of water-fertilizer management mainly focused on irrigation methods [11], irrigation amount and frequency [12], and various types of fertilizers [13–15].

Lately, an improved fertigation technique was developed that applies nutrients with water to the crops. Compared to classical fertigation, this new technique significantly increases yield, water use efficiency (WUE), and nutrient use efficiency [16–22]. For instance, compared to conventional furrow irrigation, properly controlled fertigation increases the yield of* C. annuum* and enhances WUE [11, 23]. It was experimentally shown that carefully determined fertilizer quantities could ensure high yield of* C. annuum*, at the same time reducing production costs [13]. Another study reported that compared to conventional furrow irrigation, an optimized fertigation strategy could increase the WUE of* C. annuum* by 95%, saving water by 34% and fertilizer by 20% [15].

Few studies have investigated the comprehensive influence of a water-nitrogen coupling effect on the growth, yield, and quality of* C. annuum*, and a quantitative index remains to be determined. Particularly, fruit quality as a function of the water-nitrogen ratio has rarely been reported. Therefore, the experiments presented here aimed at enhancing the utilization of water and nitrogen as well as promoting yield and quality of* C. annuum* crops. Using an automatically controlled irrigation and fertilization system, plot tests were performed in a sunlight greenhouse to optimize water and nitrogen management and make full use of water-nitrogen synergistic effects. This work provides technical support for application of automatic water and nitrogen management systems in* C. annuum* cultivation under protected conditions and offers a scientific basis for reference of high quality, high efficiency, and large-scale production.

## 2. Materials and Methods

### 2.1. Experimental Materials

#### 2.1.1. Experimental Plots

The experiments were conducted in a test area with the Guanzhong plain, China, located at 108°04′E and 34°20′N. The work was performed between April and July 2014, in a sunlight greenhouse belonging to the Key Laboratory of the Ministry of Education for Agricultural Water and Soil Engineering in Arid Area, Northwest Agriculture and Forestry University, China. The greenhouse windows face south and north, with vents at the top and southern-facing bottom of the greenhouse. The test facility has an altitude of 521 m and warm temperature due to a local semihumid climate. The annual average temperature is 13°C with annual average precipitation from 550 to 600 mm. The greenhouse measures 76 m in length, 7.5 m in width, and 2.8 m in height. Heavy soil (1% sand, 72% silt, and 27% clay) was used, with details on its physical and chemical properties shown in Table 1. The soil bulk density was measured by dry weight. Soil samples were collected from a depth between 0 and 80 cm (every 20 cm) with a ring knife (diameter: 5 cm, height: 5 cm). A small weather station (HOBO Event Logger, Onset Computer Corporation, USA) was set up inside the greenhouse. The atmospheric pressure, temperature, photo synthetically active radiation (PAR), relative humidity, and meteorological factors such as solar radiation were recorded every 10 minutes. The obtained measurements confirmed that these parameters were comparable for all plots.

#### 2.1.2. Fertigation Equipment

An online irrigation fertilizer applicator (NETAJET 3G INLINE, NETAFIM, Israel) was employed for fertigation, which can precisely apply and control fertilization based on the amount of irrigation water. The flow rate supported by this system is from 0.5 to 20 m^3^·h^−1^. Venturi-type applicators were used, equipped with an optical fertilizer meter with a flow rate capacity from 30 to 300 L·h^−1^. Both fertilizer and acid solutions can be used. In addition, the system includes electrical conductivity (EC) and pH measurement and control modules. Drip irrigation pipe (inner diameter: 8 mm) was employed using drip laterals with inline emitter, distanced at 30 cm between emitter, providing a flow rate of 2 L·h^−1^ for each emitter and pipe working pressure of 0.3 MPa.

#### 2.1.3. *C. annuum* Type and Fertilizer Used

American* C. annuum* “Marco” (*Capsicum annuum* L. var. grossum Marcomi F1) was used, as this type is particularly suitable for greenhouse production. The long, lantern shaped fruit is green when young and turns red when ripe. The fertilizer used in the experiment contains urea, calcium superphosphate (Ca(H_2_PO_4_)_2_·2H_2_O), and potassium chloride (KCl).

### 2.2. Experimental Design

Two experimental variables were studied: the amount of irrigation water and the amount of nitrogen fertilizer. Based on the reference crop evapotranspiration (ET_0_), four irrigation levels were tested, at 105% ET_0_ (W_1.05_), 90% ET_0_ (W_0.90_), 75% ET_0_ (W_0.75_), and 60% ET_0_ (W_0.60_). Based on the locally recommended nitrogen application amount of 300 kg·hm^−2^ (300 kg·hm^−2^, N_1.00_), three alternative regimes were tested with 75% N (225 kg·hm^−2^, N_0.75_), 50% (150 kg·hm^−2^, N_0.50_), and 25% (75 kg·hm^−2^, N_0.25_). The test was designed to assess all 16 possible combinations of these variables, with three identical plots for each situation, reaching a total of 48 plots. Cultivation in furrows covered with film was adopted, which is typically used by local farmers. Each ridge had a height of 25 cm and width of 75 cm. The top of the ridge was flat with spacing of 50 cm between ridges, plants were spaced at 45 cm, and rows were separated by 30 cm. Individual planting was used with a planting density of 31,000 plants per hm^2^. Each plot consists of one ridge with two plant rows. A drip lateral pipe was installed in the middle of two plant rows, so that one pipe controlled two rows. Each plot was 6.70 × 1.25 m, with a total area of 8.40 m^2^. To prevent the interaction of water and fertilizer between neighboring plots, the plots were separated by embedding plastic foil 1 m deep into the soil.

### 2.3. Irrigation and Fertilization

On January 6, 2014, seeds were soaked in water for 12 hours, followed by incubation in 1% copper sulfate solution for 5 min, and subsequently washed with water. The seeds were then placed into a thermostat for germination from 25 to 30°C for ten days. The budding seeds were sowed in a hotbed. Five weeks later, the greenhouse was disinfected as follows. For each cubic meter of soil, 5 g sulfur, 0.1 g 80% insecticide (dichlorvos, 2,2-dichlorovinyl dimethyl phosphate), and 10 g saw dust were mixed uniformly and ignited. After sealing the greenhouse overnight, the greenhouse was vented and sealed again. The elevated temperature of the greenhouse was continued for 48 hours. On April 1 and 2, the soil was prepared and basic fertilizer (phosphate) was applied. The seedlings, with 1 heart and from 8 to 12 leaves, were planted on the next day and uprooted on July 23. To ensure a proper seedling survival rate, 40 mm planting water was applied. Beginning from April 19, irrigation was conducted every five days. During the whole growth period, the total irrigation amounts for W_1.05_, W_0.90_, W_0.75_, and W_0.60_ were 263.1, 231.2, 199.4, and 167.8 mm, respectively. After transplanting the seedlings, nitrogen was applied seven times, at day 20, 40, 55, 65, 75, 85, and 95, with amounts of 13.3, 13.3, 13.3, 20, 20, 13.3, and 6.7% of the total nitrogen amount applied to the whole growth period. After cultivation for 40 days, phosphate and potassium fertilizer was applied every 15 days for a total of five times, with application of 8.5 kg·hm^−2^ of phosphate fertilizer and 25 kg·hm^−2^ of potassium fertilizer each time. During the whole growth period, plant management such as support, pruning, and thinning was performed according to local custom.

### 2.4. Measurements and Methods

#### 2.4.1. Dry Matter Content (DM)

During the experiment, three* C. annuum* plants were randomly selected from each plot on 33, 54, 66, 81, and 112 days after transplanting (DAT) and the DM content of the plants (including stems, leaves, fruit, and roots) was determined. The plant material was dried by incubation at 105°C for 30 min, followed by drying at 75°C until the weight was constant. The samples were then cooled in a dryer and weighed using a precision electronic scale. For each plot, the plant dry weight was expressed as the average of three plants, and the total biomass (t·hm^−2^) was calculated by multiplying the dry weight with the planting density.

#### 2.4.2. Chlorophyll Content

At the same time points that dry weight was determined, 0.1 g leaves were picked from randomly chosen plants for each plot. The third new leaf from the heart was selected, which grows rapidly. The amount of chlorophyll, comprised of chlorophyll a and chlorophyll b, was measured by a UV-Vis spectrophotometer (EV300PC, Thermo Fisher, USA) using an extraction method previously described [24].

#### 2.4.3. Fruit Yield

During the ripening stage, red fruits in each test plot were picked every 10 days and weighed. The yields from each pick were added together to obtain the economic yield, and this was converted to t·hm^−2^. Three plants from each plot were marked and weighed, and the average yield per plant was calculated.

#### 2.4.4. Fruit Quality

Ripe fruits with similar development characteristics were picked in each plot. The content of soluble solids in the fruits was measured as previously described [25] using an RHBO-90 hand refractometer (LINK, Co. Ltd., Taiwan China). The capsaicin content was measured by high performance liquid chromatography, and the vitamin C content was obtained by spectrometry using the molybdenum blue colorimetric method [24]. The soluble sugar content was measured by sulfuric acid anthrone colorimetry, and nitrate content was obtained using a UV-Vis spectrophotometer [19].

#### 2.4.5. Water Use Efficiency

The water content in the soil was determined with a TDR moisture meter [26] and calibrated by traditional drying method. Two days before and after the test, the water content in the soil was measured every 10 cm up to a depth of 80 cm.

The evapotranspiration (ET, mm) of the plants at various stages was calculated by a water balance based on the reference [26]. There was no precipitation in greenhouse. The deep percolation and runoff were considered negligible, since the amount of water each time was less (the maximum value was about 24.1 mm). The equation used to calculate ET is

(1) $$\begin{array}{c} ET=I-\Delta W, \end{array}$$

where *I* is the irrigation amount (mm) and Δ*W* is the water variation (mm) in the initial and final soil.

The irrigation amount *I* can be calculated as follows:

(2) $$\begin{array}{c} I=K_{c}\cdot ET_{0} \end{array}$$

where *K*_*c*_ is the crop coefficient which was based on FAO56 [27] and *K*_*c*ini_, *K*_*c*mid_, and *K*_*c*end_ were 0.60, 1.05, and 0.90, respectively.

In the greenhouse evaporation and heat transfer still occur, even in the absence of wind, since the air boundary layer is a nonneutral stable layer. ET_0_ was calculated according to the modified Penman–Monteith equation for a sunlight greenhouse, as published previously [28]. Meteorological data for calculating ET_0_ were taken from the weather station inside the greenhouse.

(3) ET0P−M=0.408ΔRn−G+γ1713ea−ed/T+273Δ+1.64γ,

where ET_0_ is the referenced crop evapotranspiration (mm·d^−1^), *R*_*n*_ is the surface net radiation (MJ·m^−2^·d^−1^), *G* is the soil heat flux (MJ·m^−2^·d^−1^), *e*_*a*_ is the saturated vapor pressure (kPa), *e*_*d*_ is the actual vapor pressure (kPa), Δ is the slope of saturated vapor pressure curve (kPa·°C^−1^), *γ* is the dry wet constant (kPa·°C^−1^), and *T* is the average temperature at 2 m (°C).

The water use efficiency WUE is calculated as follows [29]:

(4) $$\begin{array}{c} WUE=\frac{Y}{\left(ET*10\right)}, \end{array}$$

where *Y* is the yield in kg·hm^−2^.

#### 2.4.6. Partial Factor Productivity from Applied Nitrogen

The partial factor productivity from applied nitrogen (PFPN) can be calculated as follows [30]:

(5) $$\begin{array}{c} PFPN=\frac{Y}{F}, \end{array}$$

where *F* is the total mass of applied nitrogen (kg·hm^−2^).

### 2.5. Data Analysis

Statistical analysis software including Excel 2010 and SPSS Statistics 18.0 was used to analyze the experimental data. Duncan's new multiple range test method was employed for multiple comparisons. If a significant difference was observed (*P* < 0.05), Tukey HSD comparison was adopted. All figures were plotted using Origin 8.0 software.

## 3. Results

### 3.1. Influence of Water-Nitrogen Regimes on Dry Matter Content of* C. annuum*

**Figure 1:**
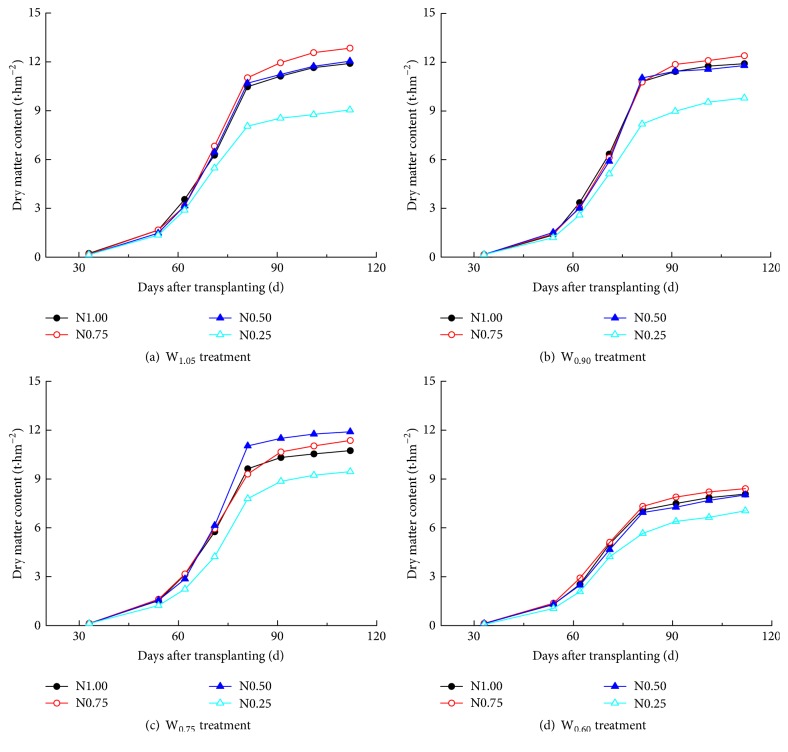
*Effects of different water and nitrogen levels on dry matter content of greenhouse sweet pepper*. Panel (a) shows treatment W_105_ corresponding to an irrigation of 105% of the reference crop evapotranspiration ET_0_, panel (b) shows treatment W_0.90_, panel (c) shows W_0.75_, and panel (d) shows treatment W_0.60_ (60% ET_0_). The black curves represent treatment N_1.00_ (100% of recommended N fertilizer, 300 kg·hm^−2^), red curves show N_0.75_, blue shows N_0.50_, and light green shows N_0.25_ (5% of recommended N fertilizer).

Growth of* C. annuum* plants was followed during the course of the experiment by determining dry matter content (DM) at various time points, as shown in Figure 1. The DM initially increased rapidly, followed by a slower increase at later stages. Fifty-four days after planting, DM starts to show increasing differences between the tested conditions. As expected, DM is affected by the amount of N supplied to the plants. According to the slopes of the curves (Figure 1), under the same irrigation regime, the increase of DM is more rapid at N_0.75_ and N_0.50_ than at N_1.00_, while N_0.25_ produced the slowest increase in dry weight.

The average DM at the highest irrigation level applied (W_1.05_) varies from 1.73 to 12.50 t·hm^−2^ at 54–112 days. For W_0.90_ this variation is 0.96–1.15 times that of W_1.05_, for W_0.75_ it is 0.91–1.05 times that of W_1.05_, and at W_0.60_ (least irrigation) the variation of average DM is only 0.78–0.76 times that of W_1.05_. Thus, compared to W_1.05_, irrigation levels W_0.90_ and W_0.75_ resulted in a more rapid DM increase in the middle and end growth stage, while W_0.60_ suppressed DM increase during the complete growth period. This indicates that, under limited water stress (irrigation levels W_0.90_ and W_0.75_), crop yield is promoted, resulting in a DM increase during the middle and end growth stage.

Keeping the nitrogen supply constant, the DM exhibits first an increase and then a decrease with increasing irrigation levels. At harvest time, the DM of N_0.75_, N_0.50_, and N_0.25_ is 1.12, 1.03, and 0.84 times that of N_1.00_, suggesting that the applied nitrogen amounts in N_0.75_ and N_0.50_ are favorable for higher DM. Thus, for both irrigation and nitrogen levels, the DM first increases followed by a decrease. At harvest time, samples with DM above 13.0 t·hm^−2^ include the combinations W_1.05_N_0.75_, W_1.05_N_0.50_, W_0.90_N_0.75_, W_0.90_N_0.50_, W_0.75_N_0.75_, and W_0.75_N_0.50_. These conditions resulted in an increase of DM of 7.77, 2.61, 9.82, 6.37, 7.22, and 3.03%, respectively, compared to the control (W_1.05_N_1.00_, DM of 12.87 t·hm^−2^) which represents local conventional irrigation and nitrogen application.

### 3.2. Influence of Water-Nitrogen Regimes on Chlorophyll Content

Chlorophyll content in the leaves was determined to provide a measure of the growth status of the plants. Chlorophyll is required for photosynthesis to produce sugars that enable growth, but a high chlorophyll content in the leaves can be disadvantageous for fruit production, when the plants favor growth of parts other than fruit.

**Figure 2:**
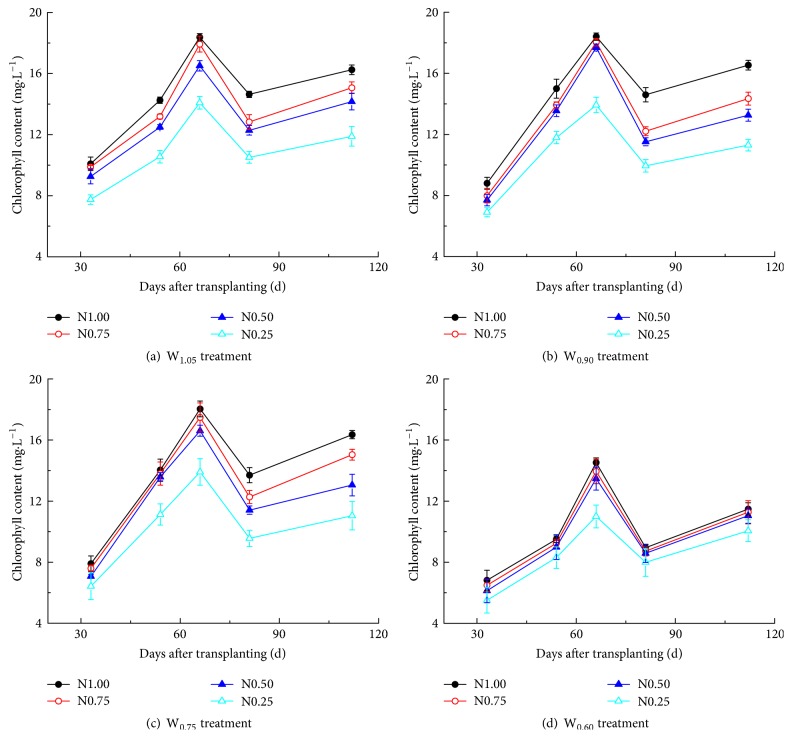
*Effects of different water and nitrogen levels on chlorophyll content of greenhouse sweet pepper*. The codes for treatment and color use are as described for Figure 1.

During growth, the overall chlorophyll content in the leaves first increased, followed by a decrease (Figure 2). Comparing conditions with the same irrigation level (individual curves within a panel) showed that the chlorophyll content increased with nitrogen levels. However, when the nitrogen level was kept constant (comparing curves with the same color), an increase in irrigation resulted first in an increase and then in a decrease of the chlorophyll content in leaves. According to the slope of the curve obtained, W_0.90_ and W_0.75_ had the largest influence on chlorophyll content compared to W_1.05_. Particularly between days 54 and 61 a rapid increase was observed. This phase corresponds to the flowering, fruiting, and reproductive stages of the plant. The products of photosynthesis are mainly reserved for growth of stems, leaves, and fruits, and these plant parts may compete with each other. Under various irrigation levels, control fertilizer level N_1.00_ resulted in a high leave chlorophyll content during the entire fruiting stage (Figure 2). Under this nitrogen supply level, a large number of small fruits developed which, after thinning, resulted in fewer nutrients being distributed to the fruits, allowing the stem and leaves to grow vigorously. In contrast, the condition W_0.60_N_0.25_ produced fewer fruits, suggesting that low water and nitrogen supplies resulted in an imbalance of resources, accelerating leaf aging and resulting in low chlorophyll content. As can be seen in Figures 2(b) and 2(c), under conditions W_0.90_N_0.75_, W_0.90_N_0.50_, W_0.75_N_0.75_, and W_0.75_N_0.50_ the chlorophyll content increased fastest to give maximum levels at day 54, indicating that limited water stress and nitrogen application can promote the allocation of nutrients to the fruits and this can accelerate fruit growth.

Towards the end of the fruiting stage the chlorophyll content of leaves was shown to increase, independent of the fertigation regime (Figure 2). This is because the perennial plant quickly develops to the next growth period after ripe fruit has been picked.

### 3.3. Influence of Water-Nitrogen Regimes on Fruit Yield and Water Usage

The influence of various irrigation and nitrogen supply combinations on economic yield, WUE, and PFPN is shown in Table 2. As can be seen, water and nitrogen levels have a significant (*P* < 0.01) impact on fruit yield, WUE, and PFPN.

Under the same irrigation condition, an increase of nitrogen level resulted in an initial increase in the economic yield and WUE, followed by decrease, while PFPN decreased (Table 2). Compared to the control water supply W_1.05_, the economic yield of W_0.90_ increased by 9.64% and that of W_0.75_ by 2.53%, while for these conditions WUE increased by 23.04 and 33.44%, respectively. In contrast, the economic yield of W_0.60_ decreased by 34.23%, with WUE increasing by 5.28%. Compared to W_1.05_, the PFPN of W_0.90_ increased by 7.22%, whereas for W_0.60_ it decreased by 35.89% (W_0.75_ showed little difference to the control). Under the same nitrogen level and an increase of irrigation, the economic yield, WUE, and PFPN initially increased followed by a decrease. Compared to nitrogen level N_1.00_, under conditions N_0.75_ and N_0.50_, the economic yield increased by 37.68 and 35.54%, respectively, while WUE increased by 35.74 and 33.67% respectively. In contrast, N_0.25_ only resulted in marginal increases of these parameters. Compared to N_1.00_, the PFPN of N_0.75_ and N_0.50_ increased by 80.46 and 166.52%. For N_0.25_ this increase was as high as 301.94%.

In combination, these results indicate that moderate water supply (W_0.90_ and W_0.75_) and limited nitrogen application (N_0.75_ and N_0.50_) promote the forming of the fruit resulting in higher yields, while improving WUE. Overly high levels of water (W_1.05_) and nitrogen (N_1.00_) supply can promote plant growth to a certain extent, but excessive growth results in reduced economic yields. Strong water stress (W_0.60_) and nitrogen stress (N_0.25_) are insufficient for optimal plant growth, leading to low DM (Figure 1) and low economic yields. Thus, compared to the control, W_1.05_N_0.75_, W_1.05_N_0.50_, W_0.90_N_0.75_, W_0.90_N_0.50_, W_0.75_N_0.75_, and W_0.75_N_0.50_ resulted in improved economic yields, with an increase of 12.39, 6.47, 20.16, 14.38, 17.13, and 15.22%, respectively. In terms of WUE, W_0.90_N_0.75_, W_0.90_N_0.50_, W_0.75_N_0.75_, and W_0.75_N_0.50_ resulted in an increase of 36.72, 30.15, 54.59, and 52.06%, respectively. Finally, again compared to the control, the PFPN increased by 60.21, 128.76, 56.18, and 130.43%, respectively. Overall, W_0.75_N_0.75_ treatment provided the optimal combination for enhanced economic yield and improved WUE and PFPN simultaneously. Similar economic yields were obtained with W_0.90_N_0.75_, W_0.90_N_0.50_, and W_0.75_N_0.50_, though the other parameters were suboptimal. Likewise, water use was also efficient in W_0.75_N_0.50_, and PFPN of W_0.90_N_0.50_ was similar to the optimal condition W_0.75_N_0.75_.

### 3.4. Influence of Water-Nitrogen Regimes on the Quality of the Produce

The influence of water and nitrogen on the content of soluble sugar, capsaicin, vitamin C (V_c_), nitrates, and soluble solids in the fruits is shown in Table 3. The tested regimes had a strong impact on the quality indexes (*P* < 0.01), and V_c_ content was also significantly affected (*P* < 0.05). Comparing increasing nitrogen levels with a constant water supply, the content of soluble sugars, V_c_, and soluble solids first increased and then decreased, while capsaicin and nitrates contents both increased. Under the same nitrogen level, an increase of irrigation resulted in a decrease in soluble sugar and nitrates content, while V_c_ content first increased and then decreased, and capsaicin and soluble solids contents both increased.

The contents of soluble sugars, capsaicin, V_c_, and soluble solids provide important indexes of fruit quality, as they determine the nutritional value and flavor. A lower nitrate content of vegetables is generally preferred, whereas greenhouse cultures have a higher nitrogen content than open-air cultures (Liao et al., 2011). Five indexes were calculated to assess the nutritional value of the produced fruit: soluble sugar (*X*_1_), capsaicin (*X*_2_), V_c_ (*X*_3_), nitrates (*X*_4_), and soluble solids (*X*_5_) using SPSS 18 software. The calculated contribution ratio for each index is as follows: soluble sugar: 42.851%, capsaicin: 28.923%, V_c_: 20.569%, nitrates: 5.779%, and soluble solids: 1.879%. The former three indexes contribute 92.342% of the total index. Thus, these three main contents were used, and the corresponding characterization values produced were *λ*_1_ = 2.143, *λ*_2_ = 1.446, and *λ*_3_ = 1.028, respectively.

By calculation, the main content can be expressed as follows:

  First main content: *F*_1_ = 0.683*X*_1_ − 0.288*X*_2_ + 0.298*X*_3_ + 0.105*X*_4_ − 0.393*X*_5_
  Second main content: *F*_2_ = −0.351*X*_1_ + 0.832*X*_2_ + 0.368*X*_3_ + 0.046*X*_4_ + 0.579*X*_5_
  Third main content: *F*_3_ = 0.431*X*_1_ + 0.436*X*_2_ + 0.986*X*_3_ − 0.084*X*_4_ + 0.038*X*_5_.

Using the ratio of each characterization value to the sum of the values as a weighing factor, a comprehensive evaluation function was established that calculated the quality of the produced fruits, given as *F* = 0.464*F*_1_ + 0.313*F*_2_ + 0.223*F*_3_. Higher scores calculated with this function indicate better fruit quality.

The results (Table 4) show that, under the same nitrogen level, W_0.75_ resulted in the highest average score, followed by W_0.90_ and W_1.05_, while W_0.60_ produced the lowest score. Under the same irrigation condition, N_0.50_ gave the highest score, followed by N_0.75_ and N_1.00_ (N_0.25_ was the lowest). These results again indicate that moderate irrigation (W_0.90_ and W_0.75_) and nitrogen (N_0.75_ and N_0.50_) levels are favorable for nutrients absorption into the fruits. Particularly, the soluble sugar and V_c_ content in the fruit can be increased using these regimes.

The conditions were ranked for the obtained scores, which placed the control at the 11th position. The top six scores were obtained with W_0.75_N_0.50_ (score value 1.64), W_0.90_N_0.50_ (1.22), W_0.75_N_0.75_ (0.79), W_0.60_N_0.50_ (0.40), W_1.05_N_0.50_ (0.35), and W_0.90_N_0.75_ (0.22). The lowest score observed (−1.16) was obtained with W_0.60_N_0.25_. This once more shows that W_0.75_N_0.50_, W_0.90_N_0.50_, and W_0.75_N_0.75_ represent favorable conditions for greenhouse culture of* C. annuum*, here assessed for parameters determined by the absorption of nutrients into the fruits.

## 4. Discussion

Adjustment of water and fertilizer supplies is the basis of optimizing agricultural practices and facility management. Proper water management and nitrogen control can improve crop growth significantly, resulting in increased economic yields, more efficient water use, and higher quality produce with lower investment costs and higher output. Conversely, poor management of water and fertilizer can lead to increased costs, wasted use of water and nitrogen resources, and negative effects on the leaf area index of crops as well as final yields [31]. Nitrogen is of particular importance, as it directly affects vegetable growth and fruit development. Proper water-nitrogen management can improve the photosynthetic assimilation of the plants and the quality of the produce [10]. The fertigation technique can provide crops with optimal supplies of water and nutrients [17, 19–21, 32]. Based on previous research, we assessed in detail the influence of water and nitrogen supplies on the growth, photosynthesis, economic yield, WUE, PFPN, and quality of* C. annuum* fruit in order to define the optimal conditions for greenhouse culture of this economically important produce.

The results have identified that conditions of water levels W_0.90_ and W_0.75_ (90 and 75% ET_0_, resp.), in combination with nitrogen levels N_0.75_ and N_0.50_ (225 and 150 kg·hm^−2^, resp.), provide an optimal window. A moderate water stress and limited nitrogen supplies promote the growth and development of fruit and result in a favorable increase of chlorophyll in the leaves, which in turn is responsible for an increase in DM. These results are in accordance with previously recorded observations [11, 23, 33]. Under the test conditions, the optimized water and nitrogen levels avoid excessive water and fertilizer use, while supporting proper growth and development. In contrast to our findings, Ayodele and colleagues concluded that the DM content of* C. annuum *positively correlates with nitrogen level supplies [34]. However, these authors tested much lower, suboptimal nitrogen levels (between 0~75 kg·hm^−2^), so that any increase will be positive. This has also been observed by others [35]. Here, we compared nitrogen levels from 150 to 225 kg·hm^−2^_,_ which covered the complete range from suboptimal to overfertilization. Candido et al. compared four nitrogen levels (0, 100, 200, and 300 kg·hm^−2^) under 100%  ET_*c*_ water level and showed that the aboveground biomass, individual fruit mass, and fruit thickness increased first and then decreased with an increase of nitrogen level [36]. Their study resulted in optimal fruit indexes at a nitrogen level of 200 kg·hm^−2^, which is comparable to our findings, though our results indicate an optimal window instead of absolute values, which is of more practical use for farmers. The optimal water supply has also been studied by Gupta and coworkers, who compared 100% ET, 80% ET, and 60% ET in combination with three NPK levels (150 : 90 : 60 kg·hm^−2^, at 100, 80, and 60%). Their results indicated that, under the same water level, the DM of* C. annuum* increased with nitrogen level, while 80% ET water level was favorable for fruit growth, giving an optimal combination of 80% ET and 80% NPK to maximize DM [33]. Our results corroborate these findings and also show the beneficial effects of a limited water stress. For sunflowers it was shown that severe drought can greatly reduce the DM, but under proper water levels, the crop growth rate can be increased by higher nitrogen levels [37]. However, for that crop the consumption of nitrogen did not change the relative growth rate and net absorption rate, consistent with findings we report here. Likewise, in previous studies concerning watermelon and muskmelon [21, 38], it was concluded that moderate water and nutrient conditions are best for vegetable growth.

Our results show that nitrogen fertilizer corresponding with 150–225 kg·hm^−2^ N in combination with irrigation conditions representing from 75% to 90% ET_0_ results in high individual plant and economic yields, while outside this range too much or too little water and nitrogen result in negative effects. This conclusion is consistent with previous works [10, 14, 39]. When zooming in on the partial factor productivity from applied nitrogen (PFPN), under the same water level, this factor decreases with an increase of nitrogen. Conversely, under the same nitrogen level, the PFPN increases first and then decreases with water supply, consistent with conclusions obtained by others [39]. Economic yields of* C. annuum* under test conditions have been reported as 27.29~65.69 t·hm^−2^ with a WUE of 14.72~32.90 kg·m^−3^ and as 29.72~46.54 t·hm^−2^ with a WUE of 7.76~10.71 kg·m^−3^ [40]. Yields as 21.01~35.30 t·hm^−2^ with a WUE of 4.7~7.9 kg·m^−3^ [23] or economic yields of 14.6~50.3 t·hm^−2^ [37], WUE of 7.8~12.3 kg·m^−3^ [41], or 4.1~6.7 kg·m^−3^ [42] have also been reported. Compared to these published results, the economic yield under optimal conditions as determined here (31.63~34.85 t·hm^−2^ with a WUE of 15.03~16.50 kg·m^−3^) had greatly improved. In part, this may be due to the type of* C. annuum* used, while the fact that experiments were conducted in a protected environment may also have helped. However, the positive effect of the online intelligent irrigation fertilizer applicator cannot be ignored, which, when set correctly, increases the utilization efficiency of both water and fertilizer.

After comprehensive consideration of economic yield, water-nitrogen use efficiency, and fruit quality, it was concluded that W_0.90_N_0.75_ resulted in the highest economic yield, with slightly reduced water-nitrogen use efficiency and quality. Although W_0.75_N_075_ resulted in the highest WUE with economic yield comparable to W_0.90_N_0.75_, its nitrogen use efficiency was poor. W_0.75_N_0.50_ resulted in lower economic yields. Compared to locally applied treatment W_1.05_N_1.00_ our optimal condition could increase yields by 15.22% with WUE improved by 52.06%. At the same time, fruits produced under W_0.75_N_0.50_ had excellent scores for contents of capsaicin, V_c_, and soluble solids, while the nitrates mass fraction was lower than the standard limit. In addition, the soluble sugar mass fraction was high, ensuing tasteful fruit.

In this study, principal component analysis was used to analyze the main factors affecting fruit quality. The results (Table 4) show that conditions W_0.90_, W_0.75_, N_0.75_, and N_0.50_, resulted in fruit of good quality. W_0.75_N_0.50_, W_0.90_N_0.50_, and W_0.75_N_0.75_ produce the top 3 rankings, while W_0.60_N_0.25_ ranks last. W_0.75_N_0.75_ has the best water-nitrogen coupling effect, and W_0.60_N_0.25_ confines the absorption of nutrients into the fruits, resulting in poor fruit quality. Other studies have also shown that proper water-nitrogen supply cannot only promote plant growth and fruit development [19] but also enhances fruit quality with no apparent reduction in yield [21, 43, 44]. These results are consistent with our work. Thus, it is plausible to improve the mass fraction of nutrients in* C. annuum* by adjusting water-nitrogen application. Moreover, an intelligent irrigation fertilizer can precisely apply and control fertilization based on the amount of irrigation water, providing accurate technical parameters for water and fertilizer integrated large-scale cultivation of* C. annuum*.

## 5. Conclusions

Experimental culture of* C. annuum* with precisely dosed water and nitrogen supplies in a greenhouse located in the northwest of China identified an optimal window between 75% and 90% ET_0_ and between 50% and 75% of conventionally used nitrogen fertilizer, resulting in an increase of economic yields of over 20%, with a simultaneous increase in DM, PPFN, and fruit quality, and an improved WUE. These insights are extremely valuable for farming practices.

## Figures and Tables

**Table 1:** Physical and chemical properties of the experimental field soil.

Soil depth (cm)	Soil bulk density (g·cm^−3^)	Field capacity (%)	Wilting point (%)	Saturated moisture (%)	pH value	Organic matter content (%)	Total N content (%)	Total P content (%)	Total K content (%)
0–20	1.46	24.4	15.2	45.3	8.03	14.5	0.08	0.06	0.17
20–40	1.57	23.8	18.2	42.0	8.15	15.7	0.08	0.05	0.14
40–60	1.48	24.7	17.6	49.0	8.20	14.3	0.06	0.04	0.14
60–80	1.45	25.2	16.0	35.2	8.20	14.0	0.05	0.02	0.12

**Table 2:** Effects of different water and nitrogen levels on sweet pepper: marketable yield and WUE.

Irrigation treatment	Nitrogen treatment	Market yield (t·hm^−2^)	WUE (kg·m^−3^)	PFPN (kg·kg^−1^)
W_1.05_	N_1.00_	22.19^de^	8.97^f^	73.96^d^
	N_0.75_	30.28^c^	11.48^de^	126.16^c^
	N_0.50_	31.51^bc^	11.95^de^	196.96^c^
	N_0.25_	24.11^de^	9.14^f^	301.32^d^

W_0.90_	N_1.00_	25.50^d^	11.00^e^	79.69^c^
	N_0.75_	34.85^a^	15.03^b^	145.19^ab^
	N_0.50_	32.48^ab^	14.01^c^	203.00^b^
	N_0.25_	25.68^d^	11.07^e^	320.95^c^

W_0.75_	N_1.00_	23.03^e^	11.52^de^	71.95^c^
	N_0.75_	32.99^ab^	16.50^a^	137.45^a^
	N_0.50_	31.63^bc^	15.82^ab^	197.67^a^
	N_0.25_	23.17^e^	11.59^de^	289.57^c^

W_0.50_	N_1.00_	15.20^g^	9.05^f^	47.51^d^
	N_0.75_	20.18^f^	12.02^de^	84.08^c^
	N_0.50_	20.84^f^	12.41^d^	130.26^c^
	N_0.25_	14.87^g^	8.86^f^	185.90^d^

Significance level (*F* value)				

Irrigation		220.77^*∗∗*^	220.77^*∗∗*^	2.60^*∗*^
Nitrogen		103.34^*∗∗*^	103.34^*∗*^	2.88^*∗*^
Irrigation × nitrogen		126.29^*∗∗*^	126.29^*∗∗*^	10.93^*∗*^

Statistical significance is shown as superscripts, with different superscripts indicating significant (*P* < 0.05) differences within a parameter under constant irrigation. At the bottom of the table, significance *F* values are indicated with ^*∗∗*^*P* value 0.001 and ^*∗*^*P* value 0.05.

**Table 3:** Effects of different water and nitrogen levels on fruit quality.

Irrigation treatment	Nitrogen Treatment	Soluble sugar‰	Capsaicin‰	V_c_‰	Nitrate‰	Soluble solids‰
W_1.05_	N_1.00_	23.55^h^	0.23^a^	25.88^de^	0.42^cd^	7.85^ab^
	N_0.75_	26.44^gh^	0.22^ab^	29.88^cd^	0.35^def^	7.40^bcdef^
	N_0.50_	30.56^def^	0.22^abc^	32.94^bc^	0.31^efg^	7.60^abc^
	N_0.25_	26.13^gh^	0.19^cdf^	26.05^de^	0.23^g^	7.50^abcde^

W_0.90_	N_1.00_	27.2^hi^	0.20^abcd^	27.81^cde^	0.45^c^	7.95^a^
	N_0.75_	32.34^d^	0.21^abc^	30.31^cd^	0.35^def^	7.55^abcd^
	N_0.50_	35.87^c^	0.21^abc^	42.24^a^	0.33^def^	7.05^defg^
	N_0.25_	27.96^efg^	0.19^cdf^	25.47^de^	0.21^g^	7.35^bcdef^

W_0.75_	N_1.00_	29.51^defg^	0.21^abc^	28.45^cde^	0.67^a^	7.25^cdef^
	N_0.75_	37.19^bc^	0.20^bcd^	36.38^b^	0.47^c^	7.20^defg^
	N_0.50_	39.27^bc^	0.21^abc^	46.53^a^	0.35^def^	7.04^defg^
	N_0.25_	31.13^de^	0.17^f^	25.39^de^	0.25^fg^	6.80^h^

W_0.60_	N_1.00_	30.80^def^	0.19^cdf^	23.67^e^	0.70^a^	6.95^fgh^
	N_0.75_	40.10^b^	0.18^df^	26.79^de^	0.57^b^	7.00^efgh^
	N_0.50_	44.67^a^	0.16^f^	30.55^cd^	0.40^cde^	6.85^gh^
	N_0.25_	31.93^d^	0.15^f^	18.91^f^	0.31^efg^	6.20^i^

Significance level (*F* value)						

Irrigation		57.86^*∗*^	15.96^*∗∗*^	24.81^*∗∗*^	26.78^*∗∗*^	27.3^*∗∗*^
Nitrogen		59.77^*∗∗*^	9.12^*∗∗*^	61.32^*∗∗*^	71.81^*∗∗*^	9.1^*∗∗*^
Irrigation × nitrogen		2.53^*∗*^	0.8	3.67^*∗∗*^	3.27^*∗*^	2.09

Significance is indicated as for Table 2.

**Table 4:** Evaluation of fruit quality under different water and nitrogen levels by multiple component analysis.

Irrigation treatment	Nitrogen treatment	Principal component			Comprehensive evaluation	Ranking
		First	Second	Third		
W_1.05_	N_1.00_	−2.256	2.670	−0.330	−0.28	11
	N_0.75_	−1.254	1.693	0.249	0.00	8
	N_0.50_	−0.744	1.590	0.886	0.35	5
	N_0.25_	−1.145	0.205	−0.959	−0.68	15

W_0.90_	N_1.00_	−1.380	1.471	−0.435	−0.28	10
	N_0.75_	−0.484	1.060	0.513	0.22	6
	N_0.50_	0.840	0.905	2.462	1.22	2
	N_0.25_	−0.784	−0.255	−0.960	−0.66	14

W_0.75_	N_1.0_	−0.333	0.669	−0.265	0.00	9
	N_0.75_	1.102	−0.075	1.369	0.79	3
	N_0.50_	1.640	0.543	3.199	1.64	1
	N_0.25_	0.277	−1.621	−1.027	−0.61	13

W_0.60_	N_1.00_	0.187	−0.811	−1.271	−0.45	12
	N_0.75_	1.447	−1.630	−0.268	0.10	7
	N_0.50_	2.349	−2.498	0.413	0.40	4
	N_0.25_	0.926	−3.434	−2.287	−1.16	16
